# Antimicrobial peptide CGA-N12 decreases the *Candida tropicalis* mitochondrial membrane potential via mitochondrial permeability transition pore

**DOI:** 10.1042/BSR20201007

**Published:** 2020-05-14

**Authors:** Ruifang Li, Jiarui Zhao, Liang Huang, Yanjie Yi, Aihua Li, Dandan Li, Mengke Tao, Youhao Liu

**Affiliations:** 1 College of Bioengineering, Henan University of Technology, Zhengzhou, Henan 450001, P.R. China; 2 School of Distance and Continuing Education, Henan University of Technology, Zhengzhou, Henan 450001, P.R. China

**Keywords:** antimicrobial peptide, C. tropicalis, CGA-N12, mitochondrial membrane potential, mPTP complex

## Abstract

Amino acid sequence from 65th to 76th residue of the N-terminus of Chromogranin A (CGA-N12) is an antimicrobial peptide (AMP). Our previous studies showed that CGA-N12 reduces *Candida tropicalis* mitochondrial membrane potential. Here, we explored the mechanism that CGA-N12 collapsed the mitochondrial membrane potential by investigations of its action on the mitochondrial permeability transition pore (mPTP) complex of *C. tropicalis*. The results showed that CGA-N12 induced cytochrome *c* (Cyt *c*) leakage, mitochondria swelling and led to polyethylene glycol (PEG) of molecular weight 1000 Da penetrate mitochondria. mPTP opening inhibitors bongkrekic acid (BA) could contract the mitochondrial swelling induced by CGA-N12, but cyclosporin A (CsA) could not. Therefore, we speculated that CGA-N12 could induce *C. tropicolis* mPTP opening by preventing the matrix-facing (m) conformation of adenine nucleotide transporter (ANT), thereby increasing the permeability of the mitochondrial membrane and resulted in the mitochondrial potential dissipation.

## Introduction

*Candida tropicalis* is the most common non-albicans *Candida* isolated from patients with candidiasis [1,2], and exhibits increased levels of fluconazole resistance [3,4]. Thus, it has become urgent to find novel and effective anti-*Candida* agents against the drug-resistant *Candida* species.

Antimicrobial peptides (AMPs) are an essential component of the defense system and have shown a broad spectrum of antimicrobial activity, high safety, low drug resistance and killing activity by multiple mechanisms [5–7]. AMPs are potent candidates for clinical infections.

Chromogranin A (CGA) exists in the secretory granules of many endocrine and neuroendocrine cells [8]. Lugardon et al. found that the highly conserved N-terminus of CGA has antibacterial activity [9]. In our previous work, we found that amino acid sequence from 65th to 76th residue of the N-terminus of CGA (CGA-N12), a more potent and stable derivative of CGA that corresponds to the N-terminal Ala55-Gln76 sequence, exhibits antagonistic activity against *Candida* species [10]. Our previous studies clarified that CGA-N12 induces mitochondrial depolarization, and cytochrome *c* (Cyt *c*) leakage, ultimately causing mitochondria-dependent apoptosis of *C. tropicalis* [11]. However, the mechanism by which CGA-N12 decreases mitochondrial potential is unclear.

The mitochondrial permeability transition pore (mPTP), a complex protein pore of the inner mitochondrial membrane (IMM) and outer mitochondrial membrane (OMM), has attracted much attention in regulating mitochondrial membrane permeability [12]. From a representative diagram of the proposed models of the mPTP, known 3-dimensional (3D) structures obtained from the Protein Data Bank (http://www.rcsb.org/pdb) [13,14], Bcl-2 associated X protein (Bax), hexokinase (HK) II, outer membrane voltage-dependent anion channel (VDAC), mitochondrial creatine kinase (mtCK), peripheral benzodiazepine receptor (TSPO), adenine nucleotide transporter (ANT) and cyclophilin-D (CyP-D) formed the mammalian mPTP complex at membrane contact sites, where VDAC and ANT formed the core pore in the OMM and IMM, respectively. ANT dimers exist in two conformations, the matrix-facing (m) and cytosolic-facing (c) conformations in the IMM. The mitochondrial permeability transition activity is determined by the orientation of the nucleotide site of ANT. The m conformation of ANT favors mPTP closure, while c conformation favors mPTP opening. When ANT is inhibited with bongkrekic acid (BA), which locks the ANT in the m conformation, it opposes mPTP opening. Mitochondrial Cyp-D bound to ANT in the matrix [15]. It was identified as a key pore opening regulator through its cyclosporin A (CsA)-sensitive interaction with the pore complex [15].

mPTP regulates the permeability of the mitochondrial membrane [13,15]. Once irreversible, mPTP opening occurs, the mitochondrial matrix osmotically swells, the OMM is damaged, and the release of Cyt *c*, leading to cell death [13,16].

Herein, the actions of CGA-N12 on *C. tropicalis* mPTP complex were investigated. We analyzed the effects of CGA-N12 on mPTP by measuring the level of mitochondrial swelling, polyethylene glycol (PEG) size across opening mPTP and Cyt *c* release.

## Materials and methods

### Microorganisms and reagents

CGA-N12 (H-ALQGAKERAHQQ-OH) was synthesized using a solid-phase method and purified by high-performance liquid chromatography. The anti-*Candida* activity of CGA-N12 was assessed by the broth micro-dilution method to define the minimum inhibitory concentration (MIC), which was 75 μM [10].

*C. tropicalis* (CGMCC2.3739) was supplied by the China General Microbiological Culture Collection Center (Beijing, China), subcultured on to Sabouraud Dextrose Agar at 28°C for 16 h, and maintained at 4°C for short-term storage.

### Isolation of mitochondria

Mitochondria were isolated from *C. tropicalis* cells following differential centrifugation procedures as previously described [17]. The isolated mitochondria were suspended in buffer C (0.6 M mannitol, 20 mM HEPES (K^+^), pH 7.35, 0.05% BSA, 0.1 mM EGTA) and stored on ice. The mitochondrial protein amount was determined by the bicinchoninic acid (BCA) method using BSA as a standard.

### Transmission electron microscopy

Ultrastructural examination of isolated mitochondrial preparations was performed using transmission electron microscopy (TEM). Mitochondria pellets were fixed in a 2.5% solution of glutaraldehyde [18] and then in a 1% solution of osmium tetroxide. After dehydrating and embedding in resin, ultra-thin sections were prepared and stained with uranyl acetate followed by lead citrate. The specimens were observed by TEM (FEI Tecnai G20, FEI, U.S.A.).

### Mitochondrial membrane potential measurement

The effect of CGA-N12 on the mitochondrial membrane potential was evaluated with JC-1 (Beyotime, Shanghai, China) followed the procedure provided by the manufacturer. Mitochondria (100 μg protein) in buffer C were pretreated with 1× MIC CGA-N12 for 1 min, and then added the diluted JC-1 working solution. After incubation at 28°C for 3 min, fluorescence at λex/λem = 485/590 nm was recorded with a fluorescence spectrophotometer (Hitachi F-4600, Hitachi, Japan) to detect the changes of mitochondrial membrane potential within 10 min. Mitochondria not treated with CGA-N12 were set as a negative control, and 1 mM ethanol was set as a positive control [19]. Each experiment was performed in triplicates.

### Mitochondrial swelling measurement

Mitochondrial swelling was measured by monitoring the absorbance at 540 nm within 10 min at room temperature as described previously [20] with minor modifications. Mitochondria (400 μg/ml protein) were suspended in 1 ml of measuring solution (300 mM mannitol, 10 mM HEPES (TEA^+^), pH 7.35, 0.2 mM EGTA, 0.5 mg/ml BSA, and 15 μg of oligomycin/mg of protein) and incubated with CGA-N12 at concentrations of 0× MIC, 0.25× MIC, 0.5× MIC, 0.75× MIC and 1× MIC. Spectra were recorded on a Shimadzu UV 2450 double-beam spectrophotometer. Mitochondria not incubated with CGA-N12 were used as a negative control, and 1 mM ethanol was used as a positive control [19]. Each experiment was performed in triplicates.

In the presence of the mPTP-opening inhibitors CsA and BA, the effects of CGA-N12 on mPTP opening were detected to determine the effect of CGA-N12 on mPTP complex [21,22]. Each experiment was repeated three times.

### Mitochondrial solute size exclusion method

The solute exclusion method was performed by measuring the pre-swollen mitochondria shrinkage caused by the osmotically active solute PEG to estimate the size of the mPTP [19,23]. Once swelling induced by 1× MIC CGA-N12 was stopped, shrinkage was promoted by the addition of different molecular weight PEGs and followed spectrophotometrically [19]. A total of 300 mOsm (milliliter/liter) PEG storage solutions in 10 mM HEPES (Na^+^), pH 7.35 was added into the measurement solution till 10% of the final volume to form an iso-osmotic solution. To get 300 mOsm PEG stock solutions, the concentrations of different molecular weight PEGs was: 199 mM for 0.4 kDa, 172 mM for 0.6 kDa, 136 mM for 1.0 kDa, 111 mM for 1.5 kDa PEG and 93 mM for 2.0 kDa. The osmotic pressure of these solutions was confirmed using a vapor pressure osmometer. Each experiment was repeated three times.

### Pre-swollen mitochondrial shrinkage measurement

The effect of CGA-N12 on mPTP opening and the action on mPTP complex were further determined by the solute exclusion of mPTP in the presence of the mPTP-opening inhibitors CsA and BA. First, 1.0 kDa PEG (PEG1000) was incubated with pre-swollen mitochondria treated by CGA-N12. The absorbance at 540 nm were continuously recorded to detect the shrinkage of the mitochondria. Each experiment was performed in triplicate.

### Cyt *c* leakage measurement

The cytosolic Cyt *c* contents of *C. tropicalis* cells post-treatment with CGA-N12 at different concentrations were measured to test the leakage of Cyt *c* from mitochondria according to the reported method [24]. *C. tropicalis* cells were incubated with 0× MIC, 0.25× MIC, 0.5× MIC, 0.75× MIC and 1× MIC CGA-N12 for 12 h at 28°C; 1 mM ethanol was set as control.

### Statistical analysis

SPSS version 21.0 (IBM, U.S.A.) was used for the statistical analysis (ANOVA and Tukey’s test). All data are presented as the mean ± standard deviation. A *P*-value <0.05 indicated a difference, *P*-value <0.01 indicated significant difference, and a *P*-value <0.001 indicated an extremely significant difference.

## Results

### Effect of CGA-N12 on the mitochondrial ultrastructure

**Figure 1:**
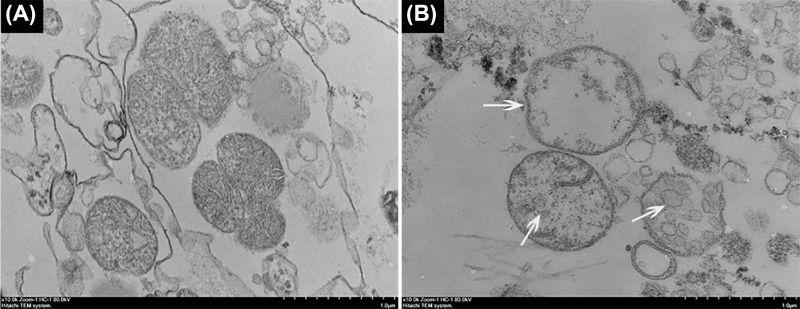
Effects of CGA-N12 on the mitochondrial ultrastructure Mitochondria were incubated without (**A**) or with 1× MIC CGA-N12 (**B**). IMM damage and cristae disappearance indicated by arrows in (B).

Isolated mitochondrial ultrastructure was observed under TEM. As shown in Figure 1, the mitochondria which were not treated with CGA-N12 maintained their integrity, showing classical ultrastructure containing well-defined outer and inner membranes, a narrow intermembrane space and compact cristae (Figure 1A). After treatment with CGA-N12, the mitochondrial ultrastructure was damaged with the disappearance of cristae and a loss of contents, but the outer membrane kept its integrity (Figure 1B). The results indicated that CGA-N12 induced the loss of the substance in the mitochondria.

### CGA-N12 induces mitochondrial membrane potential dissipation

**Figure 2:**
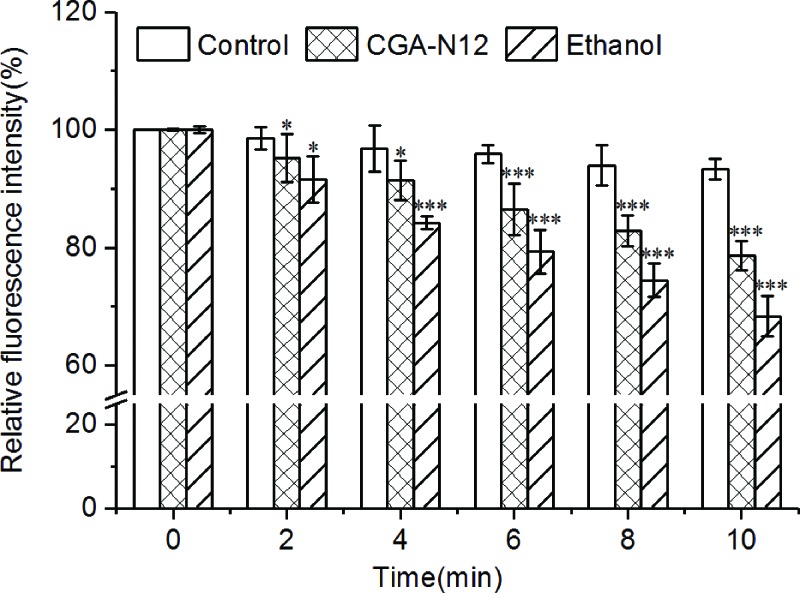
Effects of CGA-N12 on the mitochondrial membrane potential Mitochondrial membrane potential was determined by detecting the fluorescence intensity of JC-1 aggregates (λex/λem = 485/590 nm) in mitochondria. Mitochondria not treated with CGA-N12 were used as a negative control, and mitochondria treated with ethanol (1 mM) were used as a positive control. Data represent the mean ± standard deviation and *P*-values for three independent experiments (*P<0.05, ***P<0.001).

JC-1, a mitochondrial membrane potential dye, exists as aggregates in polarized mitochondria. Mitochondrial depolarization leads to the formation of JC-1 monomer [25]. We determined the fluorescence intensity of JC-1 aggregates by using a fluorescence spectrophotometer (λex/λem = 485/590 nm). The results showed that, compared with that of the control, the fluorescence intensity of mitochondria treated with CGA-N12 decreased in a time-dependent manner (Figure 2). CGA-N12 perhaps induces mitochondrial membrane potential dissipation similarly to ethanol, which could induce mPTP opening [19].

### Effects of CGA-N12 on Cyt *c* leakage

**Figure 3:**
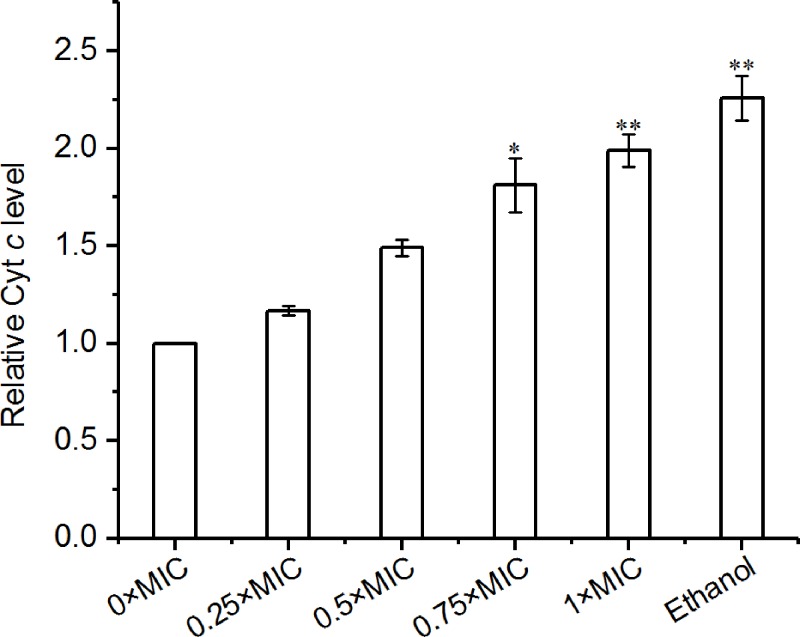
CGA-N12 induces mitochondrial Cyt *c* leakage The relative amount of reduced Cyt *c* was tested by measuring the absorbance at 550 nm to determine the content of Cyt *c* leakage. *C. tropicalis* without CGA-N12 treatment were used as a negative control, and *C. tropicalis* treated with ethanol (1 mM) were used as a positive control. Data represent the mean ± standard deviation and *P*-values for three independent experiments (*P<0.05, **P<0.01).

Cyt *c* leakage is considered an important indicator of mitochondrial permeability, which is regulated by OMM mPTP opening [26,27]. The effect of CGA-N12 on mPTP opening was determined by measuring the leakage of Cyt *c*. As shown in Figure 3, CGA-N12 induced the leakage of Cyt *c* from mitochondria in a dose-dependent manner. The results demonstrated that CGA-N12 could induce OMM mPTP opening.

### Effects of CGA-N12 on mitochondrial swelling

**Figure 4:**
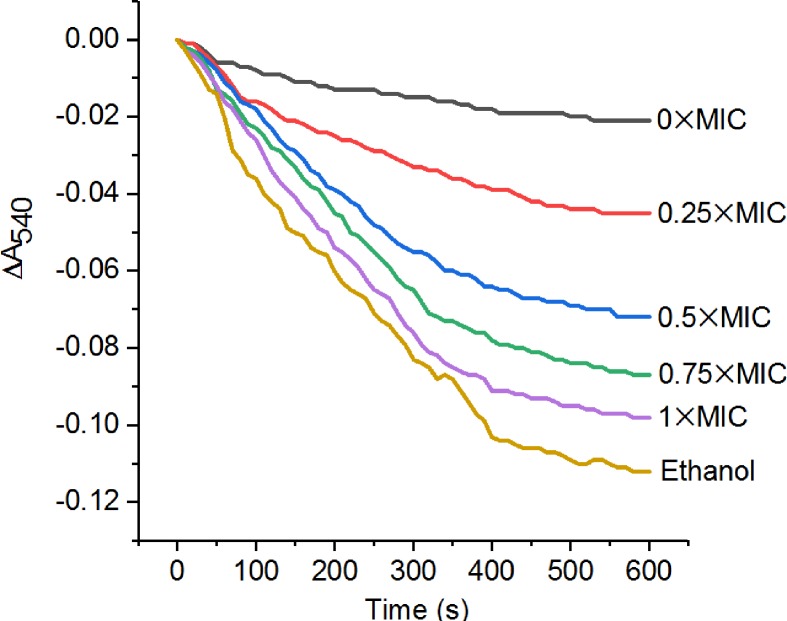
CGA-N12 induces mitochondrial swelling Each group of mitochondria (0.4 mg/ml) was treated with CGA-N12 at concentrations of 0, 0.25, 0.5, 0.75× and 1× MIC. Mitochondrial swelling was measured by monitoring the decrease in absorbance at 540 nm within 10 min at room temperature. The traces represent typical direct recordings for three independent experiments.

Mitochondrial swelling was evaluated in light of the absorbance decrease at 540 nm (A_540_) within 10 min. The effects of CGA-N12 on mitochondrial swelling are shown in Figure 4. CGA-N12 induced mitochondria swelling in a dose-dependent manner.

### Solute size exclusion properties of mPTP induced by CGA-N12

**Figure 5:**
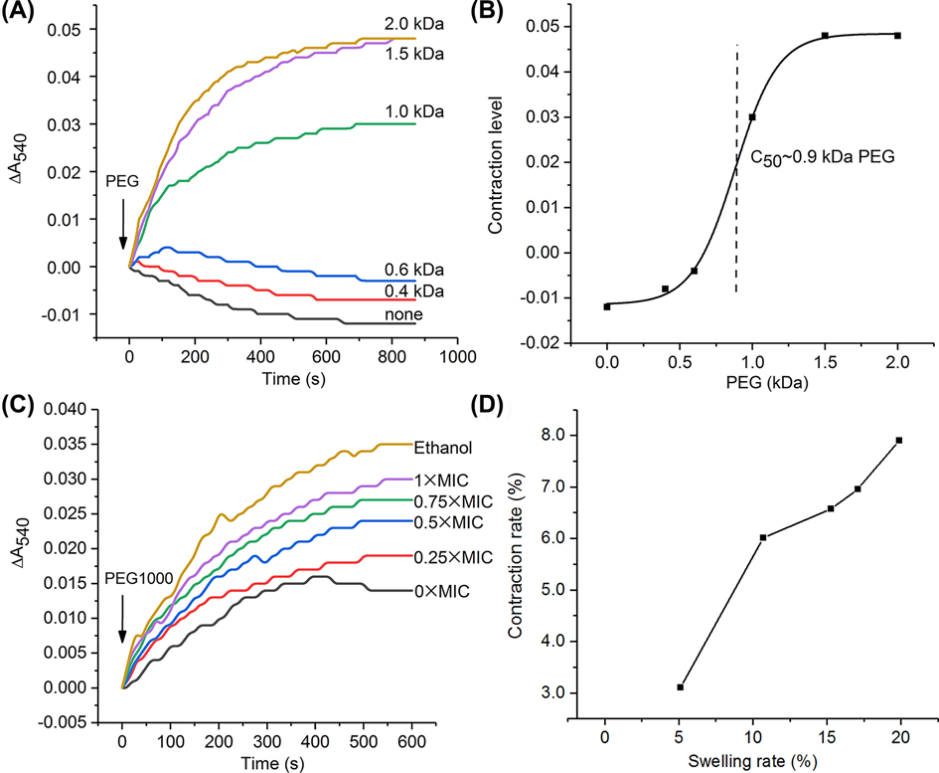
Effect of CGA-N12 on the solute size exclusion of mPTP (**A**) After the pre-swollen induced by CGA-N12 at 1× MIC, the mitochondrial contraction by 0.4, 0.6, 1.0, 1.5 and 2.0 kDa PEG were tested separately. Solutions with no PEG were set as control. (**B**) The contraction levels were plotted as a function of PEG size and fitted to a S-curve, the half-maximal contraction effect is acquired to be approximately 0.9 kDa. (**C**) Each group of mitochondria (0.4 mg/ml) was pre-swollen by CGA-N12 at 0, 0.25, 0.5, 0.75× and 1× MIC, respectively. The shrinkage of the pre-swollen mitochondria within 10 min was measured at 540 nm after the addition of PEG1000. The traces represent typical direct recordings for three independent experiments. (**D**) Plot of swelling versus shrinkage.

No shrinkage happens when PEGs could penetrate mitochondria via mPTP. The molecular weight of PEG reveals a size exclusion property of mPTP. Therefore, mPTP opening size could be estimated by the pre-swollen mitochondria contraction caused by PEG of different molecular weights. The pre-swollen mitochondria induced by CGA-N12 were treated with PEGs of various molecular weights under iso-osmotic conditions to determine their effectiveness in causing shrinkage. According to the results (Figure 5A), PEG400 (0.4 kDa) and PEG600 (0.6 kDa) readily passed through mPTP. Significant mitochondrial contraction was observed after the addition of PEG with a molecular weight more than 1.0 kDa, indicating that PEG1000 was excluded or passed through the open mPTP at a decreased rate. The contraction level induced by 1.5 kDa PEG was almost the same as that of 2.0 kDa PEG, which inferred that 1.5 kDa may be the maximum molecular mass of a PEG that can penetrate the mPTP when the yeast mPTP opens at an irreversibly high level. The relationship between the contraction levels and PEG sizes showed that the half-maximum contraction effect of swollen mitochondria induced by CGA-N12 at a 1× MIC concentration was 0.9 kDa PEG (Figure 5B).

Based on the results of solute size exclusion, PEG1000 was chosen to verify the effects of CGA-N12 on mPTP. The opening size of mPTP induced by CGA-N12 at different concentrations was determined by the swollen mitochondria contraction caused by PEG1000. The results showed that contraction degree of the swollen mitochondria increased in a concentration-dependent manner (Figure 5C). The relationship between the contraction rate and swelling rate of mitochondria treated with CGA-N12 showed that the swelling of the mitochondria induced by CGA-N12 at less than 1× MIC was nearly proportional to the shrinkage induced by PEG1000 (Figure 5D).

### Effect of CGA-N12 on mPTP complex

**Figure 6:**
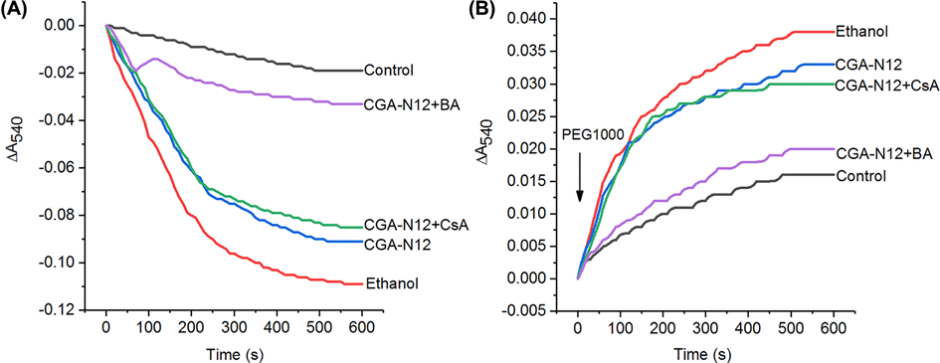
Effect of CGA-N12 on the mPTP complex proteins (**A**) The effect of mPTP-opening inhibitors on the mPTP opening induced by CGA-N12 was assessed by monitoring mitochondrial swelling. (**B**) PEG-induced shrinkage of pre-swollen mitochondria. Each group of mitochondria (0.4 mg/ml) was pre-treated with 1× MIC CGA-N12 for 1 min, and then CsA (3 μM), BA (2 μM) were added. Mitochondria not treated with CGA-N12 were used as a negative control, and mitochondria treated with ethanol (1 mM) were used as a positive control. The traces represent typical direct recordings for three independent experiments.

The effect of CGA-N12 on mPTP complex was further explored by using mPTP opening-inhibitors CsA and BA. Compared with the control, CsA treatment did not shrink mitochondrial swelling induced by CGA-N12 (Figure 6A), and there was a rapid shrinkage of the pre-swollen mitochondria caused by PEG1000 (Figure 6B). BA significantly shrank mitochondrial swelling induced by CGA-N12 (Figure 6A), and the mitochondrial shrinkage rate caused by PEG1000 was slowest (Figure 6B). The results proved that CsA could not reverse mPTP opening induced by CGA-N12, while BA could. Therefore, we speculated that CGA-N12 prevented ANT from the m conformation, while had no effect on the Cyp-D binding with CsA.

## Discussion

The mPTP complex, a nonselective channel across the IMM and OMM, is a key participant that controls mitochondrial fate and cell fate. mPTP opening is involved in various cellular processes, including membrane potential dissipation. It has been found that mPTP opening presents two distinct states: a low- and a high-conductance state. In the low-conductance state, the molecular weight cut-off value of mPTP is below 300 Da. Under such conditions, the mPTP opens transiently (‘lickering’), and mitochondrial swelling is absent [28,29]. In contrast, in the irreversible high-conductance state, the molecular weight cut-off value of the mPTP increased, allowing ions and solutes smaller than 1.5 kDa to passively diffuse across the IMM. Upon formation solutes ≤1.5 kDa in size can cross the IMM, resulting in organelle swelling and eventual rupture, a key event in necrotic cell death [30]. In the present study, the half-maximum contraction effect of swollen mitochondria induced by CGA-N12 at a 1×MIC concentration was 0.9 kDa PEG. PEG1000 passed through the mPTP post-treatment with CGA-N12. Therefore, CGA-N12 induced a high-conductance state in mPTP.

Cyt *c* in mitochondria cannot pass through the OMM unless mPTP irreversibly opens [26,27]. The mPTP opening led to a release of Cyt *c* [31]. In the present study, CGA-N12 induced Cyt *c* leakage, which indicated that CGA-N12 could open mPTP in the OMM.

Carboxyatractyloside (CAT), which stabilizes ANT in the c conformation, favors mPTP opening. While BA, which stabilizes ANT in the m conformation, favours mPTP closure [32]. In our research, the complete protection by BA against CGA-N12-induced mitochondrial swelling indicated that CGA-N12 opens mPTP by preventing ANT from the m conformation.

CyP-D controls mPTP opening by regulating its calcium sensitivity [33], while CsA is the typical inhibitor of CyP-D in mammalian cells [34]. In our research, CsA has no protective effect on the mitochondrial swelling induced by CGA-N12, although CsA is considered as a classical inhibitor of mammalian mPTP [34,35].

## Abbreviations

AMP: antimicrobial peptide
ANT: adenine nucleotide transporter
BA: bongkrekic acid
CGA: chromagranin A
CGA-N12: amino acid sequence from 65th to 76th residue of the N-terminus of CGA
CsA: cyclosporin A
CyP-D: cyclophilin-D
Cyt *c*: cytochrome *c*
IMM: inner mitochondrial membrane
MIC: minimum inhibitory concentration
mPTP: mitochondrial permeability transition pore
OMM: outer mitochondrial membrane
PEG: polyethylene glycol
PEG1000: 1.0 kDa PEG
TEM: transmission electron microscopy
VDAC: voltage-dependent anion channel
